# Persistent service even in disruptive times: an introduction to resilience engineering

**DOI:** 10.5195/jmla.2022.1359

**Published:** 2022-01-01

**Authors:** Nicole Capdarest-Arest, Sara Tompson, Lorri Zipperer

**Affiliations:** 1 ncapdarest@ucdavis.edu, F. William Blaisdell, M.D., Medical Library, University of California–Davis, Davis, CA; 2 saratifr@gmail.com, Research Solutions for Aviation and Engineering, Lawrence, KS; 3 lzipperer@ucdavis.edu, F. William Blaisdell, M.D., Medical Library, University of California–Davis, Davis, CA

## Abstract

Building resilient libraries will take energy and courage. It will take a willingness to step outside our traditional roles and engage in the messy, tough work of redefining ourselves and our institutions [1].

## BACKGROUND

Health care is a dynamic and complex business, reliant on a myriad of services, facilities, individuals, policies, and regulations to be reliably delivered—both in times of calm and in times of uncertainty. Information, evidence, and knowledge are paramount pillars of health care delivery processes such as clinical pathways, diagnostic decision-making, and quality improvement. Librarians and libraries are core to ensuring evidence access is sustained to generate reliable clinician decision-making, leadership action, staff well-being, and safe care delivery [2]. The responsibility for ensuring the resiliency of this environment *rests with the organization and its leadership*.

Resilience engineering is an organizational-level strategy that can be used to ensure service reliability. Resilience engineering encourages growth, learning, and improvement in response to unexpected disturbances, and “technology, workplace configuration, communications, and access to information all play important roles in resilience” [3]. When an academic or health care organization, and the library therein, understand and employ resilience engineering strategies, the organization as a whole can become more resilient through improved access and use of information [3]. Facilitating access and use of reliable, quality information is a core aspect of health sciences libraries, and thus librarians can be key players in enabling organizational resilience. Resilience engineering provides librarians—and library leaders, in particular—with an approach to strengthening the role of the library in the organization so that it can better provide continued and reliable information service delivery during times of disruption.

Medical librarians have long adjusted their operations in response to expected and unexpected events (e.g., pandemics, cyber-attacks, natural disasters, civil unrest, racial disparities, harmful adverse events, downsizing, mergers) in order to sustain or adjust their services [4]. Disasters, large and small, and foreseen and unforeseen changes and events will continue to occur. Disaster prevention is an important component of library management, but as prevention is not always possible, preparedness and building organizational resilience are additional ways librarians and library leaders can better adjust their outputs to changing circumstances and thus build overall organizational resilience.

Resilience is found in adaptive, complex systems, like health care and aviation, which have integral features that encourage and require resilience to protect safety. Given current societal challenges, the resilience engineering model offers a way to design systems and organizations to better accommodate and learn from disturbances.

## CONCEPT OF RESILIENCE ENGINEERING

The field of patient safety broke barriers in health care by recognizing the value and challenge in learning from other high-risk industries to make systems safer. Transferring engineering concepts to health care has been a key innovation for improving patient safety, deriving practices and processes from industries such as nuclear power, space exploration, and commercial aviation [5,6]. Resilience engineering as applied in health care environments builds upon various engineering safety tactics and models, particularly those related to failure analysis (e.g., Failure Mode Effects Analysis or “FMEA”) [7,8], as well as upon the broader concept of systems thinking [9].

One of the primary thinkers in resilience engineering, Erik Hollnagel, said: “A system is resilient if it can adjust its functioning prior to, during, or following events (changes, disturbances, and opportunities), and thereby sustain required operations under both expected and unexpected conditions” [10]. While the academic and lay literature has many discussions about personal or individual resilience [11,12], individual resilience is a different topic than resilience engineering. The importance of individual resilience and of organizational efforts to ensure the well-being of personnel during difficult times are indisputable and important; however, those areas are not the focus of resilience engineering and, therefore, will not be a topic of this commentary. Instead, we will discuss resilience engineering and its significance for recovery in response to an unanticipated disturbance, return to normal operations, and learning from the disturbance and the response to be better prepared for the next time. Resilient organizations recover quickly and return to successful, albeit altered, operations despite stumbles.

If processes are too brittle, rigid, or inflexible, if organizations are too arrogant or confident, and if learning from failure does not occur, the elements needed to quickly regain organizational stride after a disturbance are not there and the potential for continued problems remains. In these situations, responses are likely uninformed, reactionary, and unlikely to ensure successful learning from experience. To protect against the failures of rigidity, organizations can rely on librarians. Known for their responsiveness, librarians can innovate with organizational partners to ensure the purposeful sharing of information, evidence, and knowledge to facilitate returning organizational operations to a steady pace.

The elements of information, evidence, and knowledge are part of the arena in which librarians operate. The elements can be explained as follows.

- *Information:* data packaged and explained to generate understanding, facilitate communication, summarize concepts, and articulate progress (particularly during a crisis situation). For example, librarians can quickly build web portals on defined topics to ensure organizations have access to both internal and external sources of information. This is one example of why many organizations, especially academic institutions, include libraries in their disaster recovery plans.
- *Evidence:* published research results that inform actions in clinical medicine. Hypothesis-driven conclusions rooted in evidence bring robustness to decision-making. Access to evidence facilitates continued questioning during postcrisis response and management. For example, librarians can be integrated in department-level activities to provide on-demand literature review support as questions arise and postcrisis solutions are sought to implement valuable learning.
- *Knowledge:* individual experience or what one person knows based on their own experiences. For knowledge to be shared in an organization, it requires a culture that is transparent, accepting, makes time for information exchange, and encourages sharing. In certain teams or organizations (including medical libraries), sharing of knowledge has been facilitated by communication and information technologies (e.g., formal and informal professional networks, listservs, social media platforms), a networking mindset, and a desire to share. For example, even before the array of now common technologies in place, librarians established professional networks that could be used to answer questions and identify information conduits or experts who would share what they knew to provide answers to technical, reference, or research questions.

## COMPONENTS OF RESILIENCE ENGINEERING

Resilience engineering rests on four components, or capacities, that are required to enable an organization's rapid response to disturbances [3]—the abilities to monitor, respond, anticipate, and learn. These capacities are a useful way to understand resilience engineering and to begin to apply it to health care information and evidence distribution, both in libraries and in their parent organizations.

### Monitor

The monitoring of a situation, especially a developing one, is crucial for operational response [13]. Monitoring is a capacity that aligns well with the work of librarians who can connect the dots and span organizational boundaries through the work they do. Periodic environmental scans, by librarians, of the immediate organization, parent organization, and the industry can facilitate a proactive approach to unexpected events.

Misalignments and lack of goal synergies decrease resilience. Monitoring is a necessary precursor to synchronization. Synchronization helps the organization by adjusting how different roles at different levels coordinate their activities and by reducing the risk of working at cross purposes.

### Respond

The capacity to respond effectively means developing deployable and mobilizable capabilities in advance of surprises to reduce the risk of brittleness. A less brittle organization can respond more quickly and without breaking. Responding includes a range of behaviors: acting or reacting, intervening, correcting, tuning, adjusting, tweaking, trading-off, and sacrificing to achieve specific goals [3]. Fairbanks and colleagues also note that “A hallmark of resilient systems is the presence of multiple interacting goals and the active selection of goals in the face of uncertainty” [3].

The ability to prioritize actions, to triage effectively, is key to a responsive, resilient organization and its people. Response complements the capacity to anticipate as anticipation enables one to react and recover safely. A more effective response is dependent upon as much anticipation as possible.

### Anticipate

Anticipating the signs and signals of what is ahead (troubling or not) enables an organization to adapt early and reduce risks. Organizations must engineer the ability to be proactive rather than reactive. Resilient organizations are able to get out in advance of surprises and pivot to a different mode of operation when the normal mode is not going to work for the developing situation [14].

Proactive identification of relevant information, application of evidence, and connection with experts can all aid in successful anticipation of problems to prepare for ways to address them. These actions can and should influence services within libraries shaped by the expertise of librarians and library personnel. Including information services in the process of resilience engineering will enhance organizational efforts to anticipate risk. The organization will be able to establish processes that are robust yet flexible and avoid time-delaying redundancies when risks materialize, potentially filling knowledge gaps in preparation for potential problems.

### Learn

Learning is key to resilience. Understanding the tension between (ultra-safe but) brittle and resilient performance is critical in anticipating major disturbances, collapses, or accidents. Studying how other organizations have dealt with surprises, and caught and resolved them, builds an organization's own resilience. All too often, organizations do not harvest lessons from past failures and surprises.

Resilience engineering approaches can help an organization get in front of issues and learn from others. In addition to internal reflection and studying other organizations' examples of resilience, feedback is important in the Learn component. Systems thinking has a relationship to resilience engineering, in particular because systems thinking requires studying how an organization learns from itself through feedback [15]. By being prepared to respond when things unexpectedly shift, the organization gains tangible lessons from success and failure by capturing, analyzing, sharing, and using what it knows to constantly improve [16].

Librarians' skills and the services they oversee or provide can enhance each of these elements to support learning. Learning goes beyond collecting and organizing data, information, evidence, and knowledge by making sure resources are available to inform actions to be implemented to support change toward increasing system resiliency. Librarians and library leaders must look outward to demonstrate their important role in supporting the broader organization's efforts to create resiliency in their organizations. However, they also need to look inward to assure their processes and outlooks are resilient in nature. They must be ready to adapt to changes around them to effectively respond to shifts in their environment [1].

## EXAMPLE OF RESILIENCE ENGINEERING IN A HEALTH SCIENCES LIBRARY SETTING

**Case study: F. William Blaisdell, M.D., Medical Library in the COVID-19 pandemic**

### Monitor

Prior to the COVID-19 pandemic that led to widespread closures in California in March 2020, the F. William Blaisdell, M.D., Medical Library (BML) was already monitoring and preparing for disruption. BML is the medical library for the Sacramento health system campus at the University of California, Davis (UC Davis) and is part of the larger UC Davis Library.

UC Davis Library has a Library Response Team, a group that regularly meets to monitor, anticipate, respond, and learn. The Library Response Team consists of library administrators, communications specialists, and facilities supervisors from both the main (Davis) and health system (Sacramento) campus. Over the past few years, wildfires and resulting smoke have been the main disruptors to library operations and, locally on the Sacramento campus, construction-related disturbances have also been common. The Library Response Team and its members already had monitoring practices by regularly checking news and events on campus, in other libraries, and in the broader community. Prior to the massive COVID-19 shutdowns, the head of BML had already communicated with health system and library administrators in order to begin anticipating response should the need occur.

### Anticipate

Reflection and planning related to other local events that caused disruption at BML, such as the increasing wildfire events in California leading to days of hazardous air quality and rolling power outages affecting library personnel, meant that BML personnel and the Library Response Team already had established a culture oriented to responses to disruptions. The wildfire events helped us learn from and anticipate how to continue library services in these ways:

- Sustain service when personnel were not able to physically come to the library,
- Prepare and replenish emergency kits (including N95 masks for wildfire smoke) to protect personnel, and
- Write preparedness procedures and provide response training.

While better anticipating continuing operations during wildfire events, we also considered other types of events for which we could prepare. As such, we had studied and rehearsed what our library would do in certain circumstances and were also well prepared and acquainted with others in the organization who could help us during a disruptive event (e.g., library colleagues and administrators on the Davis campus, school of medicine and school of nursing administrators, local facilities management team, local campus police department).

### Respond

As the COVID-19 crisis evolved, with the UC Davis Medical Center (where BML is located) diagnosing and treating the United States' first known case of community-transmitted COVID-19 [17], BML was immediately at the heart of the crisis. In order to support frontline providers and the health professions schools, health professions schools' administrators and library leaders communicated and planned to ramp down library operations to promote safety, while keeping the BML space available to its core users.

Because we had been thinking ahead and monitoring local hospital response and management related to the COVID case, we were prepared with a response plan:

- Only allow entry to badge holders
- Shift BML staff to remote work until safety parameters and adequate personal protective equipment supplies allowed for return to on-site activities
- Communicate, plan, and support wellness for BML staff members
- Reduce seating capacity radically and reset furniture to facilitate physical distancing
- Develop, implement, and ensure a cleaning plan
- Communicate space usage expectations to the community on a regular basis
- Work with Library Communications & Marketing on communications plans and signage
- Shift all services to remote and ensure all personnel have technology to perform work remotely

As the months wore on and the situation evolved, we similarly made ramp-up and ramp-down plans, coordinated in response to onsite infections and exposures, and have thus far been able to continue operations fairly seamlessly over the last year and a half.

### Learn

Whenever there is an additional small or large shift in the disruption, we also take the time to debrief and ask one another what is working well and what could be improved. For example, when library employees came back on site and noticed traffic patterns that were less conducive to physical distancing, we came up with a barrier method and traffic direction signage to facilitate traffic flow through the library space that would intrinsically facilitate distancing. The Library Response Team continues to meet and talk through various scenarios and responses throughout the library and plan for whatever disruption might occur next.

## BUILDING ON INNOVATION

In summary, librarians, and especially library leadership, can practice and promote application of the resilience engineering model in their own environment as well as in the larger organization to foster information sharing in times of disruption. Resilience engineering works best when the larger organization's leadership is on board and fosters a culture that supports it. Librarians, with their roles as stewards, nodes, owners, adapters, appliers, and integrators of information, evidence, and knowledge in the library and beyond, model skills and behaviors that contribute to organizational resilience (Table 1).

**Table 1:** Librarian roles in broader organizations and connections to organizational resilience

**Librarian role**	**Connection to organizational resilience**	**Example**
Stewards	Individuals manage their own information resources. Librarians support this effort by ensuring resource collections are on point for clientele.	Because clinicians are able to manage some information resources, periodic feedback to/from librarians is crucial to assuring that their resources remain reliable and relevant.
Nodes	Individuals continually modify and adjust behaviors to share what they know, find evidence that supports their actions, and then draw from experience to apply it.	Librarians tap into nodes when connecting the dots in their organizations to remain informed in support of their work.
Owners	Individuals develop their own resource collections. Librarians provide services that enable that work and may also inform efforts of individuals to organize and disseminate their resources.	Contents of each individual-owned, library-curated collection are transparent, and changes are identifiable.
Adapters	Individuals constantly adapt their resource base in complex environments to adjust to change. Librarians assist with this by providing services for individuals to monitor what is happening to adjust to change in their specialty or environment.	Lessons learned from COVID-19, wildfires, and floods inform preparation for future threats for clinicians caring for patients with chronic illnesses, reduced immunity, and delayed surgeries and treatments.
Appliers	Individuals in health care exist in an information-rich workplace. They apply information, evidence, and knowledge throughout their day. Librarians can support seamless services that enable energies to be spent on application of information rather than identifying and locating what is needed.	Clinicians in training learn to work with librarians to obtain needed information and evidence such that the expectation of the alliance helps create the reality, resulting in observable changes in practice soon after best practices and treatments are published.
Integrators	Individuals regularly assemble information to connect their work with the work of others. Librarians can apply their information skills to project work to improve the monitoring and learning capabilities of teams and across the organization.	Librarians are routinely included as part of improvement committees and teams, informing proactive failure review activities to identify weakness in processes for potential crises (e.g., hospital safety processes, next pandemic, cyberattack).

To successfully implement resilience engineering and its components—monitor, anticipate, respond, learn—it is helpful for everyone in the library to be familiar with the model and its concepts. Library leadership must foster a culture of resilience and give personnel the time and space required for the work to happen. It needs to be just as okay and safe to discuss “what went wrong” as to say, “what went right.” Despite the fact that resilience engineering is not about what an individual can do, each individual has an opportunity to consider and study potential risks and possible recovery strategies.

**Figure 1:**
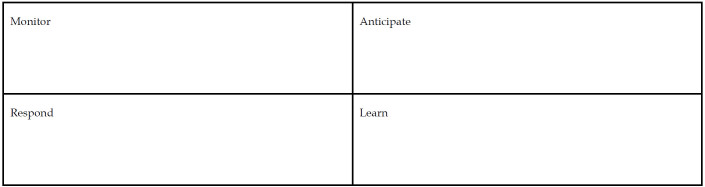
Organizational resiliency chart

You can also start by reflecting on where you stand now with regard to organizational resiliency. Use the chart in Figure 1 to make notes about what your library and organization is doing in each of the four aspects of resilience engineering. Are you particularly strong or lacking in any area? What can you do to improve any of the aspects? Are there stakeholders you can involve, technologies to consider, or discussions you can lead to improve your organizational resilience? Please share your tables with us via email to the corresponding author to facilitate spread of innovation in this area.

## Conclusion

When implemented, resilience engineering in health sciences libraries can help mitigate disruption to the libraries themselves as well as the hospitals, academic medical centers, cancer centers, societies, and corporations in which they exist and/or with whom they partner. A first approach to developing organizational resilience is to start reflecting on lessons learned from disruptions and protocols medical libraries have already put in place in response to disruptions, such as:

- Installation of shelving off the floor to protect physical materials from floods
- Cross-training staff to deal with unanticipated shortages in staff or resources (e.g., staff illness, budget cuts, Internet or technical system outages)
- LOCKSS Program [18] to preserve digital items from user error and/or cyberattacks

Additional questions to consider:

- What have you already learned from disruptions in your library?
- How can you integrate yourself into situations (e.g., rounds, morning report) where you can monitor, anticipate, respond, and learn in advance of disruptions?
- Are there others in the larger organization with whom you can connect or partner to design, demonstrate, and measure your library's commitment to organizational resilience?

As we continue into the twenty-first century, one thing is certain: disruptions will continue. Incorporating a resilience engineering model into our work can help us think and prepare more strategically with how to deal with such disturbances, as well as play a key role in communicating our value to our stakeholders as we can better articulate how our work in evidence seeking, knowledge management, information access, and knowledge dissemination can also increase the resiliency of our broader organizations.
